# Toll-Like Receptor-4 Modulation for Cancer Immunotherapy

**DOI:** 10.3389/fimmu.2014.00328

**Published:** 2014-07-25

**Authors:** Shanjana Awasthi

**Affiliations:** ^1^Department of Pharmaceutical Sciences, University of Oklahoma Health Sciences Center, Oklahoma City, OK, USA

**Keywords:** toll-like receptor 4, inflammation, immune response, cancer, immunomodulation

## Introduction

Toll-like receptors (TLRs) are evolutionarily conserved pattern recognition molecules. Since the discovery of the Toll pathway cascade (1, 2), our knowledge about the structure, function, and mechanics of TLRs in infectious and inflammatory conditions has increased remarkably. The role of TLR4 as a pathogen-pattern recognition receptor has been studied extensively. We now know that TLR4 recognizes pathogen-associated molecular patterns (PAMPs), such as Gram-negative bacterial lipopolysaccharide (LPS) and endogenous damage-associated molecular patterns (DAMPs) like fibronectin and hyaluronan, which are released during infectious and non-infectious inflammatory conditions. Some chronic infections and inflammatory conditions are known to promote carcinogenesis. For example, *Helicobacter pylori* (3) and viral hepatitis (4) infections lead to gastric and liver cancers, respectively. Also, in inflammatory bowel disease, non-infectious inflammation promotes the development of colorectal cancer (5). Evidence from recent reports suggests that increased expression and activity of TLR4 in chronic infectious and inflammatory conditions is associated with cancer progression (6–8). At the same time, additional studies suggest the protective role of TLR4 in cancer (9–14). The role of TLR4 in cancer has only recently been studied. This review article provides a brief summary of the current understanding of TLR4-signaling, its pro- and anti-cancer effects, and the therapeutic potential of TLR4 immunomodulation in the prevention and treatment of cancer.

## Ligand Recognition and Activation of TLR4

**Figure 1:**
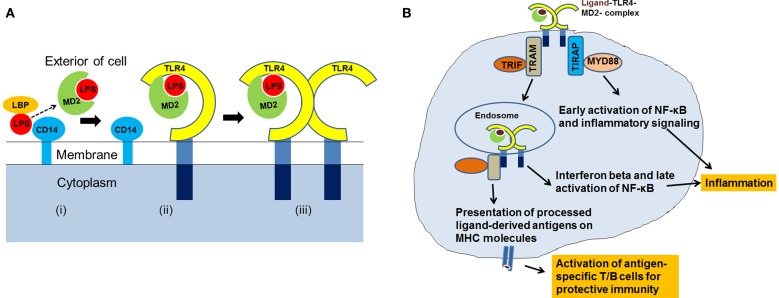
**(A)** A cartoon showing assembly of TLR4–MD2–LPS complex. The LPS is transferred to MD2 (i) Conjugation of LPS–MD2 with TLR4 (ii) then leads to dimerization of TLR4–MD2, and formation of TLR4–MD2–LPS complex (iii). **(B)** After the recognition of ligand and assembly of TLR4-complex, co-receptors: TIRAP, MYD88, TRAM and TRIF, are recruited at the intracellular level for activation of TLR4-signaling resulting into antigen-specific immune responses.

Activation of TLR4 and downstream intracellular signaling involves interaction with TLR4 ligands, dimerization, and assembly of the TLR4-complex with its adaptor and co-receptor molecules. Our understanding of TLR4-signaling is based on the results of the studies focused on the interaction of TLR4 with *Escherichia coli*-derived LPS. It has been demonstrated that the LPS binding protein (LBP) transfers the LPS to CD14 that is present in soluble form or linked to the cell surface by a glycosylphosphatidylinositol anchor. LPS is then transferred from CD14 to the myeloid differentiation (MD2) protein (6). CD14 and MD2 do not have cytoplasmic tails and are unable to transduce signals on their own. The fatty acyl chains of lipid A of LPS are integrated into the hydrophobic pocket of MD2, and negatively charged phosphate groups on the diglucosamine backbone of lipid A interact with the charged residues at the opening of the binding pocket of MD2. The LPS–MD2 interaction with TLR4 then causes dimerization of the TLR4–MD2 in 1:1 ratio and assembly of TLR4–MD2–LPS complex. The crystal structure of human TLR4–MD2–LPS complex shows the formation of an “m”-shaped oligomer made up of two molecules of TLR4 and two molecules of MD2 (15). Figure 1A provides a pictorial representation of the steps involved in the formation of the TLR4–MD2–LPS complex.

## TLR4-Signaling and Host Defense Mechanisms

After the TLR4–MD2–LPS complex formation, TLR4 signals through myeloid differentiation primary response protein (MYD88)-dependent and Toll/IL-1R domain-containing adaptor inducing interferon-beta (TRIF)-dependent pathways. Dimerization of TLR4–MD2 recruits TIRAP (Toll/IL-1R domain-containing adaptor protein) and MYD88, leading to intracellular signaling, activation of transcription factors, and production of pro-inflammatory cytokines. TLR4 also recruits additional proteins, TRIF-related adaptor molecule (TRAM) and TRIF, and induces production of type I interferons. Type I interferons are associated with immune responses elicited by T and NK cells, important effector cells of adaptive immunity (16). During the late phase of signaling, activation of TRIF can also induce NF-κB transcription factor.

Immune response to any pathogenic stimuli includes activation of innate immunity, inflammation, and adaptive immunity. TLR4-signaling can eventually lead to a multitude of cellular effects (17). It is well-established that during the innate phase of immune response, TLR4 recognizes its ligands (pathogens, PAMPs, or DAMPs), and facilitates their uptake, intracellular processing, and the inflammatory response (18–21). After the TLR4 ligands are internalized and processed, the antigens are loaded onto the major histocompatibility complex (MHC) molecules for presentation to naïve lymphocytes. Published reports support the role of TLR4 in antigen-presentation and activation of cellular and humoral immune responses (22–25). Figure 1B summarizes the recognition of ligands by TLR4, TLR4-signaling through MYD88 and TRIF, and its role in inflammation and antigen-presentation. Thus, it is apparent that TLR4 is involved directly or indirectly with different arms of the host defense system (21, 26).

## TLR4 and Cancer

TLR4 is associated with cancer in several ways. Diverse cell lines and tissue samples derived from patients with head and neck, esophageal, gastric, colorectal, liver, pancreatic, skin, breast, ovarian, cervical, and breast cancer have been shown to express increased amounts of TLR4 (27). Constitutive expression of some TLR4 genetic variants has also been linked to cancer (28–32). These characteristics are therefore being considered for their prognostic value in cancer treatment (32–34). In these scenarios of established cancer, TLR4 facilitates an environment that is suitable for continued cancer cell proliferation. Pro-cancer mechanisms could include the evasion of cancer cells from immune surveillance (35–38).

Persistent activation of TLR4-induced inflammatory signaling in chronic inflammatory conditions can also contribute to carcinogenesis (39). Experimental evidence suggests that cancer cell migration and invasion are induced by triggering of TLR4-NF-κB under inflammatory conditions (40–42). LPS-induced TLR4-signaling also promotes cancer cell survival and proliferation in hepatocellular carcinoma (43, 44). Moreover, the blockade of TLR4 by siRNA and NF-κB inhibitors decreases the invasive ability of cancer cells. Correspondingly, TLR4 silencing has been shown to decrease tumor burden in a murine model of colorectal metastasis and hepatic steatosis (45).

At the same time, published data suggest that TLR4 is required for protective immune response and killing of cancer cells. For example, TLR4-deficient mice developed more tumors after oral gavage with polyaromatic hydrocarbon 7,12-dimethylbenz(a)anthracene than did wild-type mice (46). Similarly, silencing of TLR4 increased breast cancer metastasis (47). Although mechanism is not fully understood, TLR4 can induce an efficient cancer antigen-specific cytotoxic T cell immune response (48). The cytotoxic T cells will eventually kill the cancer cells. The dynamics of the TLR4-induced immune parameters in the tumor microenvironment could be complex, and is not well studied. It is possible that TLR4 exerts pro- or anti-cancer effects, depending on the prevailing conditions in the tissue microenvironment during different phases of cancer development or metastasis.

## Currently Available TLR4 Immunomodulatory Agents

A number of immunomodulators, which target TLR4 have been developed. These modulators (antagonists or agonists) have been grouped based on their binding and sequestration of LPS, antagonizing LBP and CD14/LPS interactions, and targeting of MD2, TLR4–MD2, or TLR4.

Monophosphoryl lipid A (MPLA), a chemically modified derivative of LPS, is less toxic, and retains most of the immunostimulatory activity of LPS. MPLA serves as a TLR4 agonist. It has been approved in Europe as a vaccine adjuvant, and is a component of Hepatitis B and Human Papillomavirus Virus vaccines (49). Another lipid-based agonist, E6020 (Eisai/Sanofi Pasteur), has also been developed as a vaccine adjuvant (50, 51). Other lipid molecules are being investigated for their potential to target the CD14–LPS interaction and antagonistic activity (52). Eritoran (E5564), developed by Eisai (Tokyo, Japan), directly binds to the hydrophobic pocket of MD2, competitively inhibits LPS from binding to MD2, and prevents the dimerization of TLR4, as well as TLR4-signaling (53). TAK-242, a cyclohexene derivative, was later developed by Takeda Pharmaceuticals (Tokyo, Japan) to target the TLR4 on the cellular membrane. Both TAK-242 and Eritoran (E5564) have been investigated in clinical trials as possible treatments for sepsis (54). Ibudilast (AV4II), a TLR4 antagonist, has been shown to suppress pro-inflammatory cytokines, such as TNF-α and IL-6, in neuroinflammation (55). Antibodies that target TLR4, NI-0101, and IA6 (NovImmune, Geneva, Switzerland), are being investigated for the treatment of acute and chronic inflammation. Glucopyranosyl lipid adjuvant-stable emulsion (GLA-SE; Immune design, Seattle, WA, USA), is also being studied (http://www.clinicaltrials.gov). Although Eritoran and TAK-242 did not show efficacy for treatment of sepsis, a complicated clinical problem, studies with these modulators have clearly improved our understanding of the structural aspects of TLR4-complex formation and signaling.

## Potential of TLR4 Immunomodulation for the Prevention or Treatment of Cancer

Agents with TLR4-antagonistic activity have been shown to reduce inflammation-induced carcinogenesis by suppressing the TLR4-induced NF-κB signaling. Curcumin, the main constituent of the spice turmeric, has been found to most likely bind to MD2, thus competing with LPS (56). A number of synthetic curcuminoids, such as EF24, have also been found to have anti-inflammatory activity (57–60). Our lab recently developed TLR4-interacting surfactant protein-A (SP-A) peptide, called SPA4, which binds to TLR4 protein in complex with MD2, and is effective therapeutically in cell culture systems and in a mouse model (61, 62). In the initial studies, our results showed that the TLR4-interacting SPA4 peptide suppresses LPS–TLR4-induced migration and invasion of colon cancer cells (63). More studies are warranted to understand the mechanism of SPA4 peptide activity. Other agents, including resveratrol (64), NI-0101 antibody (65), and paeoniflorin (66), have also shown suppression of inflammation-induced carcinogenesis.

While TLR4 antagonists could help reduce progression of inflammation-induced carcinogenesis or metastasis, TLR4 agonists have been shown to induce anti-tumor immunity in patients and models of established cancer. Lipid A-based TLR4 agonists, known as OM-174 and AS15, exhibit anti-cancer effects (67–70). Incorporation of the LPS and E6020 to Paclitaxel, whole cell tumor cell vector, and Trastuzumab improved the anti-tumor immunity in mouse models (71–73). Picibanil (OK-432) targets both TLR2 and TLR4 and suppresses cancer (74). Vacchelli et al. recently published a detailed review of the ongoing clinical trials on TLR modulators, including TLR4 agonists. While the results from the ongoing clinical trials are pending, there is currently a significant emphasis on the design and development of novel TLR4 immunomodulators.

Although the potential of TLR4 immunomodulation for cancer immunotherapy has not been explored extensively, initial results from pre-clinical and clinical studies look promising. It is reasonable to imagine a TLR4 immunomodulatory agent that reduces inflammatory response, but promotes anti-tumor immunity. This could be beneficial in controlling multiple stages of cancer. Comprehensive studies are therefore needed to understand the mechanism of action of TLR4 immunomodulators in appropriate *in vitro* and *in vivo* models of cancer.

## Information about Patent Applications Pertaining to TLR4 Immunomodulation by Surfactant Protein-A (SP-A) Derived Peptides

Patent applications have been filed on the concept of TLR4-interacting SP-A peptides for immunomodulation with United States Patent and Trademark Office (USPTO), World Intellectual Property Organization, European, Canadian, and Australian Patent agencies. A patent was recently issued by the USPTO (US 8,623,832; Inventor: Shanjana Awasthi; Assigned to the Board of Regents of the University of Oklahoma, Norman, Oklahoma).

## Conflict of Interest Statement

The author declares that the research was conducted in the absence of any commercial or financial relationships that could be construed as a potential conflict of interest.
